# The Effect of ProHydrolase^®^ on the Amino Acid and Intramuscular Anabolic Signaling Response to Resistance Exercise in Trained Males

**DOI:** 10.3390/sports8020013

**Published:** 2020-01-22

**Authors:** Jeremy R. Townsend, Jaclyn E. Morimune, Megan D. Jones, Cheryle N. Beuning, Allison A. Haase, Claudia M. Boot, Stephen H. Heffington, Laurel A. Littlefield, Ruth N. Henry, Autumn C. Marshall, Trisha A. VanDusseldorp, Yuri Feito, Gerald T. Mangine

**Affiliations:** 1 Exercise and Nutrition Science Graduate Program, Lipscomb University, Nashville, TN 37204, USA; morimunej@gmail.com (J.E.M.); mdjones1@mail.lipscomb.edu (M.D.J.); shheffington@lipscomb.edu (S.H.H.); llittlefield1@lipscomb.edu (L.A.L.); henryrn@lipscomb.edu (R.N.H.); autumn.marshall@lipscomb.edu (A.C.M.); 2 Central Instrument Facility, Department of Chemistry, Colorado State University, Fort Collins, CO 80523, USA; cbeuning@rams.colostate.edu (C.N.B.); allison.haase@colostate.edu (A.A.H.); claudia.boot@colostate.edu (C.M.B.); 3 Exercise Science and Sport Management, Kennesaw State University, Kennesaw, GA 30144, USA; tvanduss@kennesaw.edu (T.A.V.); yfeito@kennesaw.edu (Y.F.); gmangine@kennesaw.edu (G.T.M.)

**Keywords:** protein supplementation, protease, muscle, leucine, protein synthesis

## Abstract

This double-blind study examined effects of a protease enzyme blend (Prohydrolase^®^) added to whey protein on post-resistance exercise aminoacidemia and intramuscular anabolic signaling were investigated in ten resistance-trained males. Participants completed 4 sets of 8–10 repetitions in the leg press and leg extension exercises at 75% of 1-repetition maximum. Participants then consumed either 250 mg of Prohydrolase^®^ + 26 g of whey protein (PW), 26 g whey alone (W), or non-nutritive control (CON) in counterbalanced order. Blood samples were obtained prior to exercise (baseline) and then immediately-post (IP), 30-, 60-, 90-, 120-, and 180-min post-exercise. Muscle biopsies were taken at baseline, 1-h (1H), and 3-h (3H) post-exercise. Phosphorylation of AKT^Ser437^ was decreased (3H only: *p* < 0.001), mTOR^Ser2448^ was increased (1H: *p* = 0.025; 3H: *p* = 0.009), and p70S6K^Thr412^ remained unchanged similarly for each condition. Plasma leucine, branch-chained amino acids, and essential amino acid concentrations for PW were significantly higher than CON (*p* < 0.05) at 30 min and similar to W. Compared to IP, PW was the only treatment with elevated plasma leucine levels at 30 min (*p* = 0.007; ∆ = 57.8 mmol/L, 95% Confidence Interval (CI): 20.0, 95.6) and EAA levels at 180 min (*p* = 0.003; ∆ = 179.1 mmol/L, 95% CI: 77.5, 280.7). Area under the curve amino acid analysis revealed no differences between PW and W. While no different than W, these data indicate that PW was the only group to produce elevated amino acid concentrations 30-min and 180-min post-ingestion.

## 1. Introduction

The maintenance and enhancement of skeletal muscle mass is increasingly relevant with wide reaching implications ranging from athletic performance to clinical outcomes. As such, resistance exercise and feeding strategies are targeted as effective interventions for the promotion of hypertrophy and preservation of muscle mass [1]. Essential amino acids (EAA) and resistance training are both potent stimulators of the mammalian target of rapamyacin complex 1 (mTORC1) signaling pathway and subsequent increases in muscle protein synthesis (MPS) rates [2,3]. Furthermore, the magnitude of p70S6k (a downstream mTORC1 signaling molecule) phosphorylation has been shown to be a proxy marker of MPS [4] that corresponds with resistance training-induced muscle hypertrophy [5,6,7]. Mitogen-activated protein kinases (MAPK) are a family of signaling proteins which have also acquired attention for their role in regulating muscular adaptations to acute and chronic resistance exercise [8,9]. Extracellular signal regulated kinase 1 and 2 (ERK1/2), c-jun NH2-terminal kinase (JNK) and p38 are MAPK proteins, along with nuclear factor kappa B (NFKB), that are activated in the muscle by the mechanical tension, cellular stress, and inflammation brought on by resistance exercise and may compliment mTORC1 in the promotion of hypertrophy [10,11,12,13]. There is evidence to suggest that these signaling pathways may be sensitive to different training protocols [10,14] and EAA availability or formulations [15,16,17]. Taken together, strategies to optimize these molecular targets are commonly sought after and implemented to augment adaptations to resistance training.

An acute session of resistance training has the ability to stimulate MPS post-exercise but at the cost of elevating muscle protein breakdown during exercise [18,19]. To promote a net balance that favors MPS, which would enable recovery and adaptation, it is important that appropriate nutrients are provided [20]. Whey protein, derived from milk, is classically recognized as the most efficient dietary simulator of MPS following resistance exercise [21,22]. This is attributable to the high leucine content and fast digestibility of whey compared to other protein sources [21,23,24]. Leucine specifically functions as a signaling molecule which possesses the ability to directly activate mTORC1 in a dose dependent manner [15,25], indicating that amino acid availability, specifically EAAs and leucine, may mediate hypertrophic and body composition adaptations to chronic resistance training [16]. To this end, various whey protein formulations (e.g., hydrolysates) have been designed to enhance protein absorption kinetics and maximize post-resistance training MPS rates [26]. Whey hydrolysates are thought to be advantageous for absorption and amino acid availability because the manufacturing process involves peptide bond cleavage that produces peptides of different lengths along with free amino acids. However, studies examining hydrolyzed whey have yielded mixed findings. Some have reported modest improvements in plasma appearance of amino acids [27], while others have found no positive effects on estimated gastric emptying [28] or aminoacidemia [29,30]. Accordingly, interest remains in finding alternative nutritional formulations that will consistently promote aminoacidemia.

One possible approach to augment plasma amino acid availability involves the usage of dietary protease enzymes [31]. Like the process of creating a hydrolysate, protease enzymes catalyze the breakdown of protein molecules into smaller peptides and amino acids for digestion and absorption. Previously, their addition to whey protein has been shown to improve in-vitro digestibility [32], support growth [33,34], and modulate the inflammatory response to eccentric exercise [35,36]. However, only one study has examined the effect of adding proteases to whey protein on aminoacidemia [31]. Oben et al. reported greater total serum amino acids and nitrogen retention in adults consuming either a low- or high-dosage of a protease supplement added to whey protein concentrate compared to controls [31]. Interestingly, the rate of serum amino acid appearance peaked at 4 h post-consumption in all groups, which does not match the generally-reported peak in amino acid concentrations with 30–60 min of whey protein ingestion [21,23,24,37]. Consequently, additional work in this area is needed to explore the efficacy of protease supplementation on amino acid availability and the subsequent effects that improved aminoacidemia may elicit on skeletal muscle.

The current study seeks to contribute additional data regarding protease supplementation in trained males. The primary outcome measure of this study was to examine the efficacy of a proprietary protease enzyme (ProHydrolase^®^, Deerland Enzymes, Kennesaw, GA, USA) on the plasma amino acid response when consumed with whey protein. The secondary outcome measure was to determine if increased amino acid availability, due to concurrent protease and whey protein ingestion, produced an augmented anabolic signaling response to acute resistance exercise compared to whey protein alone or placebo ingestion. As a tertiary outcome measure, since previous studies reported reduced inflammation in protease supplemented groups, we examined stress-activated intramuscular protein signaling responses. We hypothesized that the protease supplement would promote a larger and more rapid increase in plasma amino acids, resulting in increased phosphorylation of anabolic signaling molecules, and decrease stress-related signaling molecule activity following acute resistance exercise.

## 2. Materials and Methods

### 2.1. Study Participants

Ten resistance-trained males (24.4 ± 4.1 years, 1.79 ± 0.86 m, 92.6 ± 10.4 kg) with at least one year of resistance training experience were enrolled in this study. Specifically, all participants had recently been utilizing training protocols similar to what was prescribed in this study (3–5 sets, 6–12 repetitions, 1–2 min rest). Following an explanation of all study protocols, benefits, and risks, each participant provided their written informed consent prior to participation in this study. All participants were free of any disorders, injuries, or physical limitations which may have prevented them from being able to perform physical exercise, as determined from health and activity questionnaire. Furthermore, all participants were asked to not take any other nutritional supplements or performance enhancing drugs throughout the duration of their enrolment in the study. The study protocol was approved by the University Institutional Review Board before participant enrollment. This study was registered at clinicaltrials.gov as NCT04144114. 

### 2.2. Study Protocol

The investigation was performed as a placebo-controlled, randomized, counterbalanced, cross-over design. Participants reported to the Human Performance Lab (HPL) on 4 separate occasions over the course of the study. On the first visit, participants completed body composition assessment and were tested for maximum strength by performing a 1-repetition maximum (1RM) test for leg-press and leg-extension exercises. During the three subsequent visits to the HPL, participants performed an acute resistance exercise protocol involving the leg press and leg extension exercises. Following the acute resistance exercise protocol, participants were randomly assigned to consume one of three study treatments and began post-exercise blood draws and biopsies at predetermined intervals. Treatment order was randomized using a random number generator (https://www.google.com/search?q=random+number). 

### 2.3. Anthropometric and Body Composition Assessment

Height (±0.1 cm) and body mass (±0.1 kg) were measured for each participant using a Health-o-meter scale (Model 500 KL, Pelstar, Alsip, IL, USA) with the participants standing barefoot, with feet together, in their regular exercise attire. These data were acquired prior to all body composition and strength measures.

Body mass, non-bone fat-free mass (FFM), and body fat percentage was determined using whole body–dual energy x-ray absorptiometry (DXA) scans (Prodigy^TM^; Lunar Corporation, Madison, WI, USA). Total body estimates of percent fat and non-bone FFM (±0.1 kg) was determined using company’s recommended procedures and supplied algorithms. Daily calibrations of Quality assurance were completed prior to all DXA scans using the manufacturer supplied calibration block. All DXA assessments were completed using standardized subject positioning procedures by a single certified radiological technician.

### 2.4. Maximal Strength Testing

Participants visited to the HPL prior to the acute resistance exercise protocols to establish maximal strength (1RM) on the leg press and leg extension exercises. Each participant completed a standardized warm-up before the 1RM strength session which involved 5 min of cycling on a cycle ergometer against low resistance, 10 body-weight squats, 10 body weight walking lunges, 10 dynamic walking hamstring stretches, and 10 dynamic walking quadriceps stretches. The 1RM tests were performed using methods previously described for the leg press and leg extension exercises [38]. Two warm-up sets utilizing a resistance of approximately 40–60% and 60–80% of his estimated maximum for the exercise were performed. For both exercises, 3–4 subsequent, maximal trials were completed to determine the 1RM. A 3–5 min rest period was provided between each trial. Trials which did not meet the range of motion standards for each exercise or where correct technique was not used were discarded. Foot positioning on leg press sled as well as equipment placement (back, knee, and leg positions) for leg extension were measured, recorded, and replicated for each participant on subsequent visits. Additionally, any notable attire (such as lifting shoes), which may influence a participant’s performance, was recorded and replicated for each subsequent visit.

### 2.5. Experimental Trials

Following baseline measurements, participants reported to the lab on three additional occasions to complete each experimental condition. On the morning of each visit, participants were asked to report to the HPL fasted for 12 h and asked to refrain from all forms of moderate to vigorous leg exercise over the previous 72-h. They were also asked to report after obtaining at least 8-h of sleep and were not to have consumed any dietary supplements.

Experimental trials were completed in a balanced, randomized order with each trial being separated by a minimum of one week to warrant adequate recovery. All experimental trials were performed at the within a 1-h window to avoid diurnal variations. Due to known effects on resistance exercise performance and endocrine responses [39], ample hydration status was verified (USG ≤ 1.020 defined as euhydration) via urine samples collected upon arrival to the HPL and analyzed for specific gravity (USG) by refractometry. During each experimental trial, participants performed the same standardized warm-up routine described above for 1RM testing, followed by a lower-body resistance exercise protocol that consisted of four sets of 8–10 repetitions for the leg press and leg extension exercises at a resistance approximately equal to 75% of 1RM. Participants who were unable to complete at least eight repetitions during any set were provided with manual assistance via spotters on each remaining repetition. When this occurred, the load was reduced (~20–30lbs) on the next to set to enable 8–10 unassisted repetitions. Rest intervals between each set and between exercises were 90 s. Immediately upon completion of the acute resistance exercise bout, participants consumed their assigned supplement and completed IP assessments.

### 2.6. Supplementation Protocol

Immediately following the acute resistance protocol blood sampling, participants were required to consume one of three experimental supplementation conditions: 250 mg ProHydrolase^®^ + 26 g whey protein (PW), 26 g whey protein (W), or a non-nutritive, digestion resistant maltodextrin control (CON). Prohydrolase^®^ (Deerland Enzymes, Kennesaw, GA, USA) is proprietary proteolytic enzyme blend that can be incorporated into protein powders and other food products. All products were identical in taste and appearance and were mixed with 400 mL of water immediately before consumption. Participants were not permitted to drink water during the first 2-h post–exercise during the experimental trials. Between 120–180 min post-exercise participants were provided a bottle with 500 mL of water. Water consumption during this time was monitored, the amount consumed was recorded, and this recorded amount was replicated during subsequent trials.

### 2.7. Blood Collection, Handling, and Storage

Blood samples during the experimental trials (Baseline-180min) were drawn from a 20 g Teflon^TM^ cannula placed in an antecubital vein, which remained patent via an isotonic saline flush. Blood samples were obtained from the cannula with a plastic syringe prior to exercise (Baseline), immediately-post (IP), 30-, 60-,90-, 120-, and 180-min post-exercise. 

Blood samples were drawn into untreated (for serum collection), as well as EDTA- and heparin-treated (for plasma collection) BD Vacutainer^®^ tubes. Untreated tubes were allowed to clot for 30 min prior to centrifugation, while treated tubes were centrifuged immediately for 15 min at 1500× g at 4 °C. The resulting serum and plasma were aliquoted and stored at −80 °C until analysis. Samples were thawed only once for biochemical analysis. 

### 2.8. Muscle Biopsy Procedure

Muscle biopsies were performed via fine-needle aspiration on the vastus lateralis muscle of the participant’s left leg using a spring-loaded, reusable biopsy instrument with 14-gauge disposable needles (Argon Medical Devices Inc., Plano, TX, USA). Biopsies were obtained at baseline, one hour post-exercise (1H), and three hours post-exercise (3H). After local anesthesia (2 mL of 1% lidocaine) was administered into the subcutaneous tissue, a small incision was made and a coaxial introducer needle was placed perpendicular to the muscle, piercing the fascia [40]. The biopsy needle was inserted through the coaxial introducer and a muscle sample was obtained by engaging the activation button on the biopsy instrument, which unloaded the spring and engaged the needle to gather a tissue sample. Muscle samples were then removed from the needle using a sterile hypodermic needle, placed in a cryotube, immediately frozen in liquid nitrogen, and stored at −80 °C for later analysis.

### 2.9. Plasma Insulin Analysis

Circulating plasma concentrations of insulin were assessed via enzyme-linked immunosorbent assays (ELISAs) and a FLUOstar Omega spectrophotometer (BMGLabtech, Ortenberg, Germany) using commercially available kits (ALPCO, Salem, NH, USA). To minimize interassay variance, all samples were thawed once and ran in duplicate in the same assay performed by a single investigator. The coefficient of variation for insulin was 11.1%. 

### 2.10. Plasma Amino Acid Analysis 

#### 2.10.1. Materials

Cell free solid 20 amino acid (AA) mix of non-labeled standards, STD-AA, (ULM-7891, Lot: PR-28676) and ^13^C, ^15^N—isotopically labeled AA internal standard mix, IS-AA, (CNLM-6696: U-13C, 97–99%+; U-15N, 97–99%, Lot: PR-22794) were purchased from Cambridge Isotope Labs, Inc. (CIL) (Tewksbury, MA, USA) and stored at 4 °C. LC/MS grade acetonitrile, water, ammonium acetate, and formic acid were purchased from Fisher-Scientific (Hampton, NH, USA).

#### 2.10.2. Sample Preparation

The extraction solution for all plasma samples consisted of 3:1 ACN:H_2_O with 10 mM ammonium acetate and 0.1 (v)% formic acid including the IS-AA mix, see Tables S1 and S2 for stock and final STD-AA/IS-AA concentrations. Plasma samples were extracted in a 1:9 ratio of plasma:extraction solution, vortexed for 10 s, and centrifuged at 15,000× g for 10 min [41]. Supernatant was transferred to a 96 well plate (Waters), then sealed with clear film covers to prevent evaporation.

A study pool quality control (SPQC) sample was created by mixing an equal volume aliquot from each unknown sample. All calibration standards (C1–C7) were prepared in a composite plasma sample taken from healthy volunteers for matrix matching (detailed calibrator preparation information in supplement information). A true blank was prepared by extracting calibrator plasma matrix using the same extraction solvent with no IS-AA mix. Calibrators C1–C4 were used as the quality control samples.

#### 2.10.3. LC/MS Data Acquisition and Processing

Hydrophilic interaction chromatography (HILIC) separation of calibrators and experimental samples was performed on a Waters Acquity H-Class UPLC instrument equipped with a quaternary pump and an Acquity UPLC^®^ BEH Amide column (2.1 × 100 mm, 1.7 µm particle size, part 186004801) including a Van Guard™ UPLC BEH Amide pre-column (2.1 × 5 mm, 1.7 µm particle size, part 186004799). Column temperature was 35.0 °C, while the autosampler was kept at 10.0 °C; injections were 1 µL, and solvent flow rate was 0.4 mL/min. The UPLC was in line with a Waters Xevo TQD (triple quadrupole) Z*spray* ESI (electrospray ionization) mass spectrometer, which was controlled by MassLynx software (version 4.2). The mass spectrometer was operated in ESI-positive mode, at a capillary voltage of 1.50 kV, with a desolvation temperature of 350 °C, a desolvation gas flow of 650 L/h, a cone source gas flow of 0 L/h, and a source temperature of 150 °C. Gradient separation was performed using the following solvents channel A: water, B: acetonitrile, C: 500 mM ammonium acetate in water, and D: 5 (V)% formic acid in water; gradient conditions are outlined in Table A1 [41]. The column was equilibrated for at least 4 min in the starting conditions before use and after every 8 samples an acetonitrile solvent blank was run. 

Multiple reaction monitoring (MRM) was used to quantify amino acid content in the samples. The quantifier ion (first transition listed) and qualifier ion (second transition listed) for each amino acid, their retention time, cone voltage, and collision energy are given in Table A2. The IS-AA MRM transitions, cone voltages, and collision energies are given in Table A3. The IS-AAs had identical retention times as the non-isotopically labeled STD-AAs. Dwell times were automatically selected at 0.005 s. Results from histidine and cysteine quantification were not reproducible, they are omitted from these tables. 

Following acquisition, data files were imported into Skyline Targeted Mass Spec Environment for processing [42]. Retention time matching was manually inspected, and a seven-point bilinear regression weighted 1/x^2^, calibration was created for each AA (0.2 to 26.2 µg/mL global range; see supplemental information for calibration range of individual AA) [43]. The AA concentration of each sample was calculated relative to the peak area for isotopically labeled internal standards.

#### 2.10.4. Quality Control of LC/MS Data

When data processing in Skyline was completed, data were further analyzed (Microsoft Excel) to determine if the plate met pre-set quality control (QC) criteria (Table S3). The following criteria were required to meet QC: R^2^ value above 0.95 with a minimum of 6 points in the calibration curve, 75% of the non-zero calibrators within ±15% of the theoretical concentration, except lower limit of quantitation (LLOQ) should be within ±20% [44]. Additional details on the calibration range, limit of quantitation and QC results are provided in the supplemental information.

### 2.11. Intramuscular Signaling Analysis

Prior to homogenization, muscle samples were kept on ice and a lysis buffer with protease inhibitors (EMD Millipore, Billerica, MA, USA) was added to each sample at a rate of 500 µL per 10 mg of muscle tissue. Muscle was homogenized via sonication (Branson, Danbury, CT, USA) and a Teflon pestle. Homogenized muscle samples were then placed on a plate shaker (Thermo Fisher Scientific Inc., Waltham, MA, USA) for 10 min at 4 °C and then centrifuged at 10,000× *g* for 5 min to remove insoluble proteins. For each sample the supernatant was aspirated and used for further analysis.

Quantification of the phosphorylation status of proteins specific to anabolic signaling pathways were completed using a multiplex signaling assay kit (EMD Millipore, Billerica, MA, USA) and a multiplex plate reader (MAGPIX®; Luminex, Austin, TX, USA) according to manufacturer’s recommendations. Previous work reported comparable results between mTOR proteins in this signaling multiplex and immunoblotting or protein activity assays [45]. Samples were analyzed for phosphorylation of AKT^Ser474^, mTOR^Ser2448^, p70S6K^Thr412^, ERK 1/2^Thr185/Tyr187^, JNK^Thr183/Tyr185^, p38^Thr180/Tyr182^, and NFKB^Ser536^. Total protein quantification was conducted using a detergent compatible (DC) protein assay kit (Bio-Rad, Hercules, CA, USA). Homogenized samples were diluted prior to being loaded and results are reported as fluorescence intensity based upon the multiplex assay results relative to total protein content. To eliminate inter-assay variance, all tissue samples were thawed once and analyzed in duplicate in the same assay run by a single technician. The average coefficient of variation was 9.4% for the phosphor–protein analysis.

### 2.12. Dietary Logs

Participants were coached to maintain their usual dietary intake in the days preceding the acute resistance exercise trials. Throughout the 24 h before first experimental trial, each participant was additionally instructed to record everything they consumed in a precise manner. For subsequent trials, participants were asked to replicate the content, quantity, and timing of their daily diet during the 24 h prior to the experimental trial. Further, instructions were given to each participant not to eat or drink anything (except water) within 12 h of arriving to the HPL for study trials.

### 2.13. Statistical Analysis

Prior to analysis all data was assessed to ensure normal distribution, homogeneity of variance and sphericity. Non-normally distributed data were transformed using the natural log (LN) and if sphericity was violated, a Greenhouse Geisser correction was applied. General Linear Model (GLM) repeated measures analyses (time × group) were used to compare all trials (PW, W, CON) for all variables except total amino acids (TAA). When appropriate, follow-up analyses included 1-way repeated measures analysis of variance (ANOVAs) and Tukey post hoc comparisons. Area under the curve (AUC) was also calculated for changes in plasma insulin and amino acid response using a standard trapezoidal technique, and a one-way ANOVA was used to examine differences among groups. An alpha level of *p* < 0.05 was considered statistically significant for all comparisons. Using an effect size 0.24, a power of 0.80 ([beta] = 0.20) and a significance level ([alpha]) of 0.05, it was determined that ≥9 participants were required to detect significance based on changes in plasma leucine [27]. In addition, the partial eta squared statistic was calculated for effect size for all dependent variables, with 0.01, 0.06, and 0.14 were interpreted as small, medium, and large effect sizes, respectively [46]. 

Additionally, after transformation, one variable did not meet the assumption of normality (i.e., TAA) and was examined separately across trials and time via a generalized linear mixed model with maximum likelihood estimation and an autoregressive-heterogenous repeated covariance to account for the dependent relationships existing between time points. Succeeding any significant F-ratio, precise differences were additionally evaluated by applying corrections to confidence intervals (CI) using the Bonferroni procedure. All data are reported as mean ± SD and all statistical analyses were performed using SPSS version 22.0 (IBM SPSS Statistics for Windows, Version 22.0. Armonk, NY: IBM Corp.).

## 3. Results

Participant characteristics are presented in Table 1. While 12 participants were originally recruited to participate in this study, 10 participants were included for analysis. Two participants were unable to complete the study protocol due to unresolvable scheduling conflicts. All study products were well tolerated and there were no reported adverse effects from the supplementation protocol. All participants were adequately hydrated (USG ≤ 1.020) prior to each trial, and no significant differences were noted between trials for baseline USG (*p* = 0.348). There were no significant differences (*p* = 0.983) in completed exercise volume (sets × load × repetitions) between treatments (PW: 33217.0 ± 6593.2; W: 33142.5 ± 6572.8; CON: 33634 ± 6065.5). 

### 3.1. Plasma Insulin

**Figure 1:**
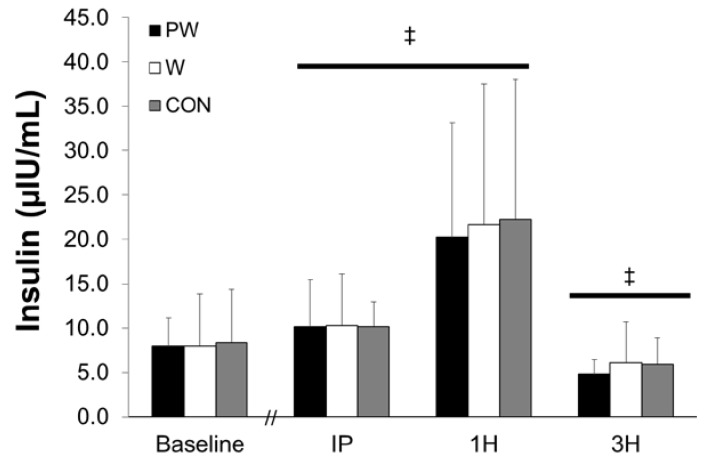
Insulin concentrations following resistance exercise and consumption of three study treatments over 3 h. Groups: PW = Prohydrolase + Whey; W = Whey; CON = Non-nutritive control. Time points: IP = Immediately post-exercise; 1H = One hour post-exercise; 3H = Three hours post-exercise. ‡ Horizontal bar indicates a main effect of time for all 3 groups. Values represent means ± SD.

There was no group x time interaction (F = 0.050, *p* = 0.96, η^2^ = 0.004) for plasma insulin concentrations (Figure 1). A main effect for time observed for plasma insulin with an increase in concentrations observed at IP (*p* = 0.001) and 1H (*p* < 0.001), while decreased concentrations were seen at 3H (*p* = 0.001). Further, there were no differences observed between groups for insulin AUC (F = 0.063, *p* = 0.939). 

### 3.2. Plasma Amino Acids

**Figure 2:**
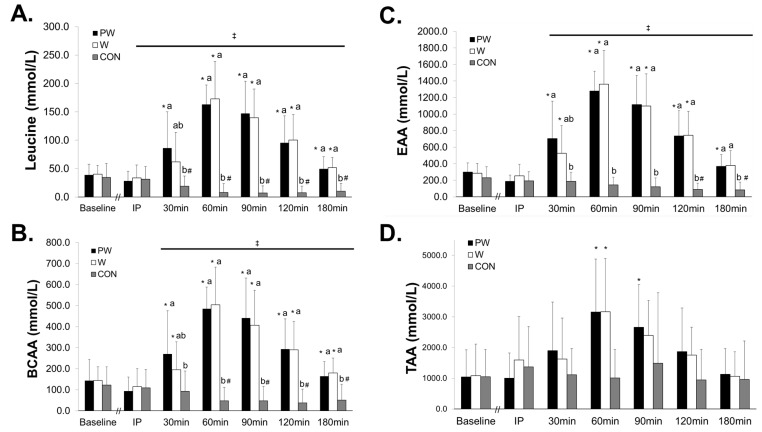
Plasma leucine (**A**), branched-chain amino acids (BCAA) (**B**), essential amino acids (EAA) (**C**), and total amino acids (TAA) (**D**) following consumption of three study treatments over 180 min. Groups: PW = Prohydrolase + Whey; W = Whey; CON = Non-nutritive control. ‡ Horizontal bar indicates a main effect of time for all 3 groups. * Elevated from respective group’s IP measurement. # Decreased from respective group’s IP measurement. Labelled means without a common letter differ at that time point. Values represent means ± SD.

There was a significant group x time interaction found for plasma leucine concentrations (F = 16.38, *p* < 0.001, η^2^ = 0.548) with PW and W groups producing significantly greater concentrations (*p* < 0.05) at 60–180 min compared to CON (Figure 2). At 30 min post-ingestion, PW was significantly elevated (*p* = 0.013) compared to CON, while not different than W (*p* = 0.508). Additionally, PW was the only group which was significantly elevated (*p* = 0.007) compared to IP. Plasma leucine was significantly decreased (*p* < 0.05) in CON from 30–180 min following ingestion in comparison to IP. Leucine AUC concentrations were significantly greater (*p* < 0.001) in PW and W compared to CON, with no differences in AUC seen between PW and W (*p* = 0.990).

A significant group x time interaction was found for plasma BCAA concentrations (F = 16.15, *p* < 0.001, η^2^ = 0.545) with PW and W groups producing higher BCAA concentrations (*p* < 0.05) than CON at 30–180 min. At 30 min post-ingestion, PW was significantly elevated compared to CON (*p* = 0.037), while no different than W (*p* = 0.523). Furthermore, plasma BCAA concentrations were significantly decreased (*p* < 0.05) in CON from 60–180 min post-ingestion compared to IP. Plasma BCAA AUC concentrations were significantly greater (*p* < 0.001) in PW and W compared to CON, with no differences in AUC seen between PW and W (*p* = 0.872).

A significant group x time interaction was found for plasma EAA concentrations (F = 14.01, *p* < 0.001, η^2^ = 0.509) with PW and W groups producing higher EAA concentrations (*p* < 0.05) than CON at 30–180 min. At 30 min post-ingestion, PW was significantly elevated compared to CON (*p* = 0.005) while no different than W (*p* = 0.467). At 180 min, PW was the only group significantly elevated (*p* = 0.003) compared to its respective concentrations at IP. Furthermore, plasma EAA concentrations were significantly decreased (*p* < 0.05) in CON from 120–180 min post-ingestion compared to IP. Plasma EAA AUC concentrations were significantly greater (*p* < 0.001) in PW and W compared to CON, with no differences in AUC seen between PW and W (*p* = 0.991).

The mixed model analysis revealed a significant group x time interaction for TAA (F = 3.40, *p* = 0.001). Plasma TAA were elevated from IP concentrations at 60 min and 90 min for PW (60 min: 2157.9 ± 992 mmol/L (95% CI: 385.2, 3930.6), *p* = 0.005; 90 min: 1660 ± 979.3 mmol/L (95% CI: 495.9, 2824.9), *p* < 0.000) and at 60 min for W (1571.2 ± 776.2 mmol/L (95% CI: 115.0, 3027.4), *p* = 0.025) with no changes being observed during CON. The one-way ANOVA revealed no differences in TAA AUC concentrations between groups (*p* = 0.147).

### 3.3. Anabolic Signaling

**Figure 3:**
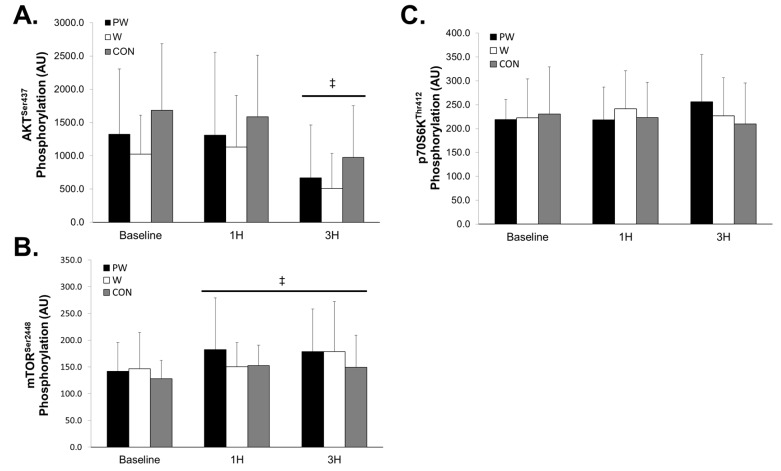
Phosphorylation of AKT^Ser437^ (**A**), mTOR^Ser2448^ (**B**), and p70S6K^Thr412^ (**C**) following resistance exercise and consumption of three study treatments over 3 h. AU = Arbitrary Units. Groups: PW = Prohydrolase + Whey; W = Whey; CON = Non-nutritive control. Time points: IP = Immediately post-exercise; 1H = One hour post-exercise; 3H = Three hours post-exercise. ‡ Horizontal bar indicates a main effect of time for all 3 groups. Values represent means ± SD.

There was no group x time interaction for AKT phosphorylation (F = 0.206, *p* = 0.934, η^2^ = 0.015; Figure 3A). There was a main effect for time for decreased phosphorylation compared baseline at 3H (*p* < 0.001). There was no group x time interaction for mTOR phosphorylation (F = 0.561, *p* = 0.691, η^2^ = 0.040; Figure 3B). However, there was a main effect for increased phosphorylation at 1H (*p* = 0.025) and 3H (*p* = 0.009) compared to baseline. There was no group x time interaction for p70S6K phosphorylation (F = 0.860, *p* = 0.494, η^2^ = 0.060; Figure 3C). Additionally, no main effect for time was seen for p70S6K phosphorylation (F = 0.194, *p* = 0.901, η^2^ = 0.004).

Additionally, there were no group × time effects seen for targets in the MAPK signaling cascade JNK (F = 0.991, *p* = 0.420, η2 = 0.068), ERK 1/2 (F = 0.0206, *p* = 0.934, η^2^ = 0.015), or p38 (F = 0.448, *p* = 0.771, η^2^ = 0.032). There was a main effect for time with increased phosphorylation at 1H for JNK (*p* = 0.001), and decreased phosphorylation at 3H for ERK 1/2 (*p* = 0.022) with respect to baseline. Furthermore, there was no group × time interaction for p38 phosphorylation (*p* = 0.362), nor was there a main effect for time observed for NFKB phosphorylation (*p* = 0.233).

## 4. Discussion

The present study sought to examine the effect of a protease supplement added to whey protein on acute amino acid availability and muscle protein signaling following heavy lower-body resistance exercise in trained males. To accomplish this, comparisons were made between participants after they ingested 250 mg of ProHydrolase^®^ with whey protein, whey protein only, and a non-nutritive control drink. In contrast to our secondary and tertiary outcome measure hypotheses, we observed no differences between groups in phosphorylation of signaling proteins AKT, mTOR, p70S6K, JNK, ERK 1/2, p38, and NFKB. With regards to our primary outcome measure, it appears that regardless of protease administration, leucine, BCAA, EAA, and TAA AUC values were no different between PW and W. Whilst these data did not confirm our hypothesis, it was partially substantiated in that PW was the only group with elevated leucine, BCAA, and EAA at 30 min compared to a control drink. Further, the protease supplement allowed for increased leucine concentrations at 30 min and elevated EAA concentrations at 180 min compared to IP concentrations, while W and CON were not increased at this time point.

Thus far, only one other study has investigated the effects of digestive proteases on protein absorption rate in healthy participants. Oben et al. [31] reported increased TAA concentrations, TAA AUC, and improved nitrogen balance when participants consumed 50 g of whey protein concentrate with a patented protease supplement derived from the Aspergillus fungus. These findings are contrary to our data as we observed differences between treatments for leucine, BCAA, and EAA at 30 min post-ingestion and no difference in AUC between protein groups. Interestingly, Oben et al. [31] indicated higher TAA concentrations in the protease groups from 1–4 h post ingestion with TAA concentrations peaking in all groups 4-h post-ingestion. However, a wealth of literature reports blood amino acid concentrations peaking much sooner (~45 min–1 h) following whey protein consumption apart from a mixed meal [17,23,37,47,48,49]. It is possible the formulation of whey concentrate utilized in that study was unique compared to other literature and the whey product used in the current investigation which accounts for the delay in amino acid absorption. Furthermore, as protease additives or whey protein modification theoretically should merely affect the rate of absorption [50], rather than increase nitrogen balance which was found in Oben et al. [31]. Due to the differences in amino acid responses and due to the fact that we did not measure nitrogen balance in the present study, we take caution in reconciling our findings with Oben et al. [31]. Nevertheless, the results of the present study indicate that 30-min following consumption, W produced similar amino acid concentrations to the PW and CON, while PW administration resulted in elevated plasma amino acid concentrations compared to CON.

Our results also indicate that while the PW improved AA concentrations (~25–28%) at 30 min over W, there were no significant differences in any amino acid parameter between PW and W at any time point. This is in line with other work demonstrating limited potential to further improve the absorption rate of whey protein by modification [28,29,30]. Studies examining hydrolyzed proteins generally report improved leucine, BCAA, or EAA amino acid values at various time points without significant differences from an unmodified whey treatment at any time point [28,29,51]. While other investigations have yielded no benefit of hydrolysis [30]. Although there were no differences in AUC between protein supplemented groups, quicker uptake in leucine, BCAA, and EAA may indicate improved digestion and absorption during this initial window following ingestion. Though outside the scope of this study, a practical application of protease supplementation may be for improving pre- or peri-workout protein digestion, thus, athletes may benefit from future investigations concerning timing of protease supplementation with whey or other protein blends.

The results of our study indicated the resistance exercise bout produced increased mTOR phosphorylation at 1H and 3H post-resistance training with decreased AKT phosphorylation at 3H post-exercise. Further, we observed no effects of protein supplementation (PW or W) on the regulation of AKT, mTOR, or p70S6K at any measurement time point. In contrast, previous studies designate resistance exercise and EAA supplementation as potent stimulators of downstream proteins in the mTOR signaling pathway, particularly p70S6K [17,26,52,53]. However, the responses have been inconsistent in resistance-trained individuals. In trained males, two heavy lower body resistance training protocols produced reduced AKT phosphorylation at 5-h post-exercise with no change in p70S6k status [54] while others have reported increased mTOR phosphorylation at 1H and 3H post-resistance training [52,53]. Furthermore, others have shown no effect of protein supplementation on p70S6K or other downstream mTOR effectors post-resistance exercise [47,48,55]. As signaling responses often differ based on participant training status [56,57], it is possible that a novel stimulus would have produced increased p70S6K phosphorylation. Although there is evidence in untrained males which describes no differences in the anabolic signaling or myofibrillar synthetic rate response to resistance exercise following the consumption of various protein types, despite greater AA concentrations experienced in some groups [24,37]. In the present study, we utilized a whey protein dose of 26 g (0.28/kg) which is above the amount (0.24 g/kg) which has been suggested to maximize MPS in recovery from resistance exercise [58]. Therefore, while we observed a more rapid rise in EAA in our study, it is not surprising that no differences in anabolic signaling were observed. It is possible that protease supplementation may prove more beneficial lower quality proteins (e.g., pea, soy) are consumed in contrast to high quality milk proteins where notable differences in digestibility and absorption are key variables which delineate protein quality [21,59]. Moreover, examination of the mTORC1 pathway provides, at best, a surrogate marker of myofibrillar protein synthesis and a “snapshot” of cellular interactions at one point in the recovery timeline. Therefore, future studies utilizing direct measurement of MPS would provide better insight to the anabolic potential of protease supplementation. 

Alterations in the phosphorylation status of ERK 1/2 and JNK have been suggested to contribute to muscle remodeling following strenuous exercise and regulate hypertrophic training adaptations [8]. Our data revealed that ERK 1/2 phosphorylation was decreased at 3H post-exercise and with elevations in JNK phosphorylation occurring at 1H. This is in partial agreement with previous work reporting increased ERK 1/2 and JNK phosphorylation following resistance exercise of varying intensities [10,13,14] with elevations generally seen close to the cessation of exercise. We observed no effect of protease supplementation on either ERK 1/2 or JNK activity despite varying plasma amino acid concentrations between our 3 treatments. This is not startling news as previous reports have demonstrated no effect of protein feeding alone on MAPK signaling [60,61]. As protease supplementation has been shown to alter skeletal muscle function following the mechanical strain of resistance exercise [35,62], we postulated it may influence multiple signaling pathways related to stress. Nevertheless, our findings provide additional support indicating minimal influence of amino acid availability on MAPK signaling and suggest that protease supplementation may not alter the MAPK signaling response to resistance exercise in trained males.

Independent of protein supplementation, some studies have investigated the effects of the physiological effects of protease supplementation surrounding exercise stress. Protease supplementation has shown to reduce muscle soreness [36] while improving recovery of muscle function and blunting inflammation following an eccentric bout of downhill running [35,36]. Other work found a small improvement for recovery of isometric forearm flexion following eccentric exercise with protease treatment [61] and possible improvement of subjective fatigue in cyclists during a competitive race [63]. Interestingly, these investigations suggest reduced muscle damage as the underpinning mechanism for improved recovery, whereas only one reported effect of protease administration on indirect blood markers of inflammation or muscle damage [35]. Intramuscularly, the regulation of p38 MAPK and NFKB can be indicative of inflammatory and remodeling processes [57,64] with concomitant activation of p38 and the NFKB signaling cascade reported following resistance training [65]. In the present study, there were no differences in p38 or NFKB phosphorylation between groups. We also found no change in p38 phosphorylation in response to resistance exercise which is in disagreement with studies in trained [14,65] and untrained [37,61,66] males describing increased post-exercise p38 phosphorylation. In support of our findings, Kudrna et al. [13] reported no change in p38 phosphorylation following 3 different (30%, 70%, and 90% of 1RM) high-velocity squat routines in males with at least 2 years of squat training experience. Further, in untrained males NFKB phosphorylation compared to baseline or control conditions [38]. Therefore, while the resistance exercise protocol was sufficient to increase mTOR signaling, it may not have provided a potent stimulus to trigger MAPK and NFKB activity. Future work surrounding these signaling pathways in untrained and trained participants would provide additional mechanistic insight to previous reports of decreased soreness and improved muscle function following unaccustomed exercise and protease supplementation. 

## 5. Conclusions

As we found no differences between treatments regarding intramuscular signaling, this study adds to the body of knowledge suggesting aminoacidemia is not the sole mediator of the anabolic signaling response to resistance exercise. Practically, athletes and active individuals can likely benefit from consuming a variety of protein sources surrounding exercise. Future work investigating protease supplementation with other protein types (e.g., casein, pea, soy), which are limited by digestion and absorption in comparison to whey, would provide additional insight to athletes as to the practical benefit of protease supplementation. Furthermore, this manuscript adds to the body of knowledge describing changes in MAPK and NFKB signal transduction proteins in trained males in the presence or absence of post-exercise nutrients. The results of this study suggest that the addition of a proprietary protease enzyme blend to whey protein results in an earlier and more pronounced aminoacidemia in resistance trained males compared to a control drink, while whey was no different than control at this time point (30 min). 

## Figures and Tables

**Table 1:** Participant characteristics.

Variable	Values
n	10
Age (years)	24.4 ± 4.1
Height (m)	1.79 ± 0.86
Weight (kg)	92.6 ± 10.4
Non-bone fat-free mass (FFM) (kg)	69.6 ± 6.8
Body fat (%)	20.9 ± 3.1
Leg press 1RM (kg)	424.5 ± 71.7

Data presented as mean ± SD.

## Supplementary Materials

The following are available online at https://www.mdpi.com/2075-4663/8/2/13/s1, Amino acid analysis Experimental Methods. Table S1: Amino acid content as weight mass percent and stock concentrations of STD-AA or IS-AA mixtures, Table S2: Final concentrations of standard amino acid (STD-AA) and isotopically labeled amino acid internal standards (IS-AA) when diluted with plasma, Table S3: Average values per compound across all plates for R2, Working range, QC accuracy, and other figures of merit.

## Appendix A

**Table A1:** The gradient used in the chromatographic separation of amino acids where the solvents used were; A: water, B: acetonitrile, C: 500 mM ammonium acetate in water, and D: 5 (V)% formic acid in water.

Time, min	% A	% B	% C	% D
0	11	85	2	2
6.0	11	85	2	2
6.1	16	80	2	2
10.0	26	70	2	2
12.0	36	60	2	2
12.1	11	85	2	2
18.0	11	85	2	2

**Table A2:** The amino acid multiple reaction monitoring (MRM) transitions quantifier ion (first transition listed) and qualifier ion (second transition listed), chromatographic retention times, cone voltages, and collision energies.

Amino Acid	Mass AA, g/mol	MRM Transition	Retention Time, min	Cone Voltage, V	Collision Energy, eV
Ala	89.1	90.1 > 44.0	6.50	15	9
Arg	174.2	175.1 > 70.1	11.06	25	20
		175.1 > 116.0	11.06	25	19
Asn	132.1	133.0 > 74.0	9.05	20	15
		133.0 > 88.0	9.05	20	10
Asp	133.1	134.0 > 74.0	10.57	20	14
		134.0 > 88.0	10.57	20	14
Gln	146.2	147.0 > 84.0	8.80	15	15
		147.0 > 102.0	8.82	15	19
Glu	147.1	148.0 > 84.0	9.64	18	16
		148.0 > 102.0	9.63	18	15
Gly	75.1	76.0 > 30.0	8.00	20	7
		76.0 > 48.0	8.02	20	7
Ile	131.2	132.0 > 69.0	2.99	15	15
		132.0 > 86.0	2.97	15	15
Leu	131.2	132.0 > 30.0	2.69	15	15
		132.0 > 86.0	2.70	15	15
Lys	146.2	147.0 > 84.0	11.42	20	15
		147.0 > 130.0	11.42	20	13
Met	149.2	150.0 > 104.0	3.38	16	10
		150.0 > 133.0	3.36	16	10
Phe	165.2	166.0 > 120.0	2.62	20	15
		166.0 > 103.0	2.62	20	20
Pro	115.1	116.0 > 70.1	4.04	15	15
		116.0 > 43.1	4.02	15	15
Ser	105.1	106.0 > 60.0	8.83	20	9
		106.0 > 42.0	8.85	20	15
Thr	119.1	120.0 > 74.0	7.55	15	11
		120.0 > 56.0	7.51	15	11
Trp	204.2	205.0 > 146.0	2.66	15	18
		205.0 > 188.0	2.68	15	18
Tyr	181.2	182.0 > 136.0	4.24	20	15
		182.0 > 165.0	4.23	20	19
Val	117.1	118.0 > 72.0	3.90	18	10
		118.0 > 55.0	3.90	18	10

**Table A3:** The isotopically labeled amino acid internal standards MRM transitions, cone voltages, and collision energies. IS-AAs had identical retention times as their non-isotopically labeled counterparts.

Amino Acid	IS-AA MRM Transition	Cone Voltage, V	Collision Energy, eV
Ala	94.1 > 47.1	15	8
Arg	185.1 > 75.1	25	20
Asn	139.0 > 77.0	20	15
Asp	139.0 > 77.0	20	14
Gln	154.0 > 89.0	15	15
Glu	154.1 > 107.1	18	16
Gly	79.1 > 32.1	20	7
Ile	139.0 > 92.0	15	15
Leu	139.0 > 92.0	15	15
Lys	155.2 > 137.1	15	14
Met	156.2 > 109.2	16	10
Phe	176.2 > 128.1	20	15
Pro	122.1 > 75.1	15	15
Ser	110.1 > 63.1	20	9
Thr	125.1 > 78.1	15	11
Trp	218.0 > 155.0	15	18
Tyr	192.2 > 145.2	20	15
Val	124.1 > 77.1	18	10
