# Supplementary material for: The Effect of ProHydrolase® on the Amino Acid and Intramuscular Anabolic Signaling Response to Resistance Exercise in Trained Males
Source: Sports (Basel). 2020 Jan 22;8(2):13. doi: 10.3390/sports8020013 (PMC7077235; doi:10.3390/sports8020013)
Supplement: Supplementary file 1 [file sports-08-00013-s001.pdf]

sports

Supplementary Information

The Effect of ProHydrolase® on the Amino Acid and Intramuscular Anabolic Signaling Response to Resistance Exercise in Trained Males

Jeremy R. Townsend [1,*], Jaclyn E. Morimune [1], Megan D. Jones [1], Cheryle N. Beuning [2], Allison A. Haase [2], Claudia M. Boot [2], Stephen H. Heffington [1], Laurel A. Littlefield [1], Ruth N. Henry [1], Autumn C. Marshall [1], Trisha A. VanDusseldorp [3], Yuri Feito [3] and Gerald T. Mangine [3]

[1] Exercise and Nutrition Science Graduate Program, Lipscomb University, Nashville, TN 37204, USA
[2] Central Instrument Facility, Department of Chemistry, Colorado State University, Fort Collins, CO 80523, USA
[3] Exercise Science and Sport Management, Kennesaw State University, Kennesaw, GA 30144, USA
* Correspondence: jrtownsend@lipscomb.edu

Experimental Methods

Stock Standards Preparation

The stock STD-AA (48.4 mg) and IS-AA (66.7 mg) standard solutions were dissolved in 10.00 mL of 50/50 ACN/H_2O with 0.1 (v)% formic acid, vortexed, and filtered with a 0.2 µm filter. These standard solutions were clear, slightly brown in color, and stored in a −20 °C freezer. Table S1 lists the amino acid mass percentages by weight from each mixture and the stock concentrations. Results from histidine and cysteine quantification were not reproducible, they are omitted from the table.

Table S1. Amino acid content as weight mass percent and stock concentrations of STD-AA or IS-AA mixtures.

Amino Acid	STD-AA mass %	IS-AA mass %	Stock STD-AA ug/mL	Stock IS-AA ug/mL
Ala—alanine	5.9	7.5	245	404
Arg—arginine	2.8	6.6	116	355
Asn—asparagine	5.1	4.7	212	253
Asp—aspartic acid	8.2	7.9	341	425
Gln—glutamine	5.1	5.4	212	291
Glu—glutamic acid	0.7	6.9	29.1	371
Gly—glycine	4.5	3.7	187	199
Ile—isoleucine	1.3	6	54.0	323
Leu—leucine	5.8	10.4	241	560
Lys—lysine	9.8	8.2	407	441
Met—methionine	3.6	1.5	149	80.7
Phe—phenylalanine	5.5	7.3	228	393
Pro—proline	4.1	1.9	170	102
Ser—serine	2.9	2.3	120	124
Thr—threonine	11.8	4.1	490	221
Trp—tryptophan	3.3	3	137	161
Tyr—tyrosine	4.9	4.5	203	242
Val—valine	7.4	6.6	307	355

Calibrator Preparation

Calibrator solutions (C1–C7) of STD-AA were prepared with serial dilution (concentration for individual amino acids in Table S2). C1 was the stock STD-AA solution listed in Table S1 and C2–C4

are serial dilutions starting with C1 of 500 μL in 1000 μL. C5 was 100 μL of C1 in 1000 μL and C6–C7 are serial dilutions starting from C5 of 500 μL in 1000 μL. To create the calibrator standards in the plasma matrix, 40 μL of the C1–C7 calibrator solutions was added to 600 μL of IS-ES, and 80 μL of plasma matrix and their final concentrations are listed in Table S2. Post preparation of the calibrator stock solutions, the extraction solution was appropriately diluted to account for the solvent introduced in the calibrator samples such that the experimental samples would have the same final concentration of internal standards.

Table S2. Final concentrations of standard amino acid (STD-AA) and isotopically labeled amino acid internal standards (IS-AA) when diluted with plasma. STD-AA concentration is for the calibration series (C1–C7) and IS-AA is for both the calibration series and the experimental samples.

Amino Acid	STD-AA C1 ug/mL	STD-AA C2 ug/mL	STD-AA C3 ug/mL	STD-AA C4 ug/mL	STD-AA C5 ug/mL	STD-AA C6 ug/mL	STD-AA C7 ug/mL	IS-AA ug/mL
Ala	13.6	6.81	3.40	1.70	1.36	0.681	0.340	2.36
Arg	6.46	3.23	1.61	0.808	0.646	0.323	0.162	2.07
Asn	11.8	5.88	2.94	1.47	1.18	0.588	0.294	1.48
Asp	18.9	9.46	4.73	2.36	1.89	0.946	0.473	2.48
Gln	11.8	5.88	2.94	1.47	1.18	0.588	0.294	1.70
Glu	1.61	0.81	0.404	0.202	0.162	0.081	0.040	2.17
Gly	10.4	5.19	2.60	1.30	1.04	0.519	0.260	1.16
Ile	3.00	1.50	0.750	0.375	0.300	0.150	0.075	1.88
Leu	13.4	6.69	3.35	1.67	1.34	0.669	0.335	3.27
Lys	22.6	11.3	5.65	2.83	2.26	1.13	0.565	2.58
Met	8.31	4.15	2.08	1.04	0.831	0.415	0.208	0.471
Phe	12.7	6.34	3.17	1.59	1.27	0.634	0.317	2.29
Pro	9.46	4.73	2.36	1.18	0.946	0.473	0.237	0.597
Ser	6.69	3.35	1.67	0.836	0.669	0.335	0.167	0.722
Thr	27.2	13.6	6.81	3.40	2.72	1.36	0.681	1.29
Trp	7.61	3.81	1.90	0.952	0.761	0.381	0.190	0.942
Tyr	11.3	5.65	2.83	1.41	1.13	0.565	0.283	1.41
Val	17.1	8.54	4.27	2.13	1.71	0.854	0.427	2.07

Detailed Data Processing

All the data for each plate was imported into Skyline at the same time, with individual plates each correlated with an individual data file. Once all compounds were checked and retention times were adjusted as needed, the plate was rescored to update peak areas for further analysis. This process was repeated until retention times no longer needed adjustment.

The calibration curve for this assay used a bilinear regression which allows for more data points at the cost of greater processing power, and the $1/x^2$ weighting is most appropriate for bioanalytical LC MS/MS [1]. The bilinear regression calculates a linear response above a turning point, a flat response below the turning point, and the turning point (TP) may be considered the limit of detection (LOD) for bilinear calibration curves. Points were removed from the calibration curve starting with the least accurate samples until the R^2 value per compound was 0.95 or greater, and the points remaining were within ±20% of the theoretical concentration. The LOD was calculated as concentration of the blank plus 3 times the standard deviation of the blank external standards. The limit of quantitation (LOQ) was determined to be the lowest concentration value that fit within the parameters of the maximum bias and maximum coefficient of variation (CV). The max LOQ bias is the maximum allowed difference from the calibration curve and was set at 15%, while the max LOQ CV is the maximum allowed CV of the peak areas of the set of internal standards (IS) with a specified concentration and was set to 15%.

The LLOQ was determined to be the highest value between the Skyline determined LOD, LOQ, and TP, and is usually the LOQ. For the QC samples with known concentrations, they were divided into 4 levels, Lower limit QC (LLQC), Lower QC (LQC), Medium QC (MQC), and High QC (HQC).

At least 50% of the QC's per level and 67% of the overall QCs needed to be within 15% of the theoretical concentrations. 50% of the sample pool QCs needed to be within ±50% of the average concentration of the sample pool QC. Each analyte per plate was analyzed separately to determine if it can be accurately quantitated on the plate, then these analyte values were averaged to determine plate data quality.

Table S3. Average values per compound across all plates for R^2, Working range, QC accuracy, and other figures of merit.

Amino Acid	R^2	Working Max (µg/mL)	Working Min (µg/mL)	% of Known QC within 15%	# of Points Used	LOD (µg/mL)	LOQ (µg/mL)	TP (µg/mL)
arginine	0.988	6.5	0.5	82%	11	<0.01	0.49	0.0002
lysine	0.990	22.6	1.3	89%	12	<0.01	1.35	0.19
glycine	0.981	10.4	2.1	49%	8	<0.01	2.13	0.22
alanine	0.984	13.6	0.7	76%	12	<0.01	0.73	0.26
serine	0.989	6.7	0.9	55%	8	<0.01	0.92	-0.07
proline	0.985	9.5	1.1	73%	10	<0.01	1.12	0.29
valine	0.991	16.5	1.4	73%	10	<0.01	1.45	0.43
threonine	0.988	26.2	1.9	76%	12	<0.01	1.86	0.60
isoleucine	0.990	5.4	0.4	82%	11	<0.01	0.40	0.08
leucine	0.991	12.3	0.5	94%	13	<0.01	0.50	0.01
asparagine	0.988	11.9	1.3	62%	10	<0.01	1.29	0.22
glutamine	0.981	11.8	4.9	19%	6	0.44	4.93	0.51
glutamic acid	0.991	1.6	0.2	71%	11	<0.01	0.20	0.03
aspartic acid	0.985	18.9	1.8	79%	10	0.26	1.84	0.48
methionine	0.995	8.3	0.7	79%	11	<0.01	0.65	0.01
phenylalanine	0.984	12.7	4.9	37%	8	<0.01	4.89	0.27
tyrosine	0.980	11.3	2.7	35%	8	0.06	2.67	0.01
tryptophan	0.988	7.6	1.3	57%	9	<0.01	1.31	0.18

References

1. Gu, H.; Liu, G.; Wang, J.; Aubry, A.-F.o.; Arnold, M.E. Selecting the correct weighting factors for linear and quadratic calibration curves with least-squares regression algorithm in bioanalytical LC-MS/MS assays and impacts of using incorrect weighting factors on curve stability, data quality, and assay performance. *Anal. Chem.* **2014**, *86*, 8959–8966.
